# Long COVID: An opportunity to focus on post-acute infection syndromes

**DOI:** 10.1016/j.lanepe.2022.100540

**Published:** 2022-11-01

**Authors:** The Lancet Regional Health – Europe

Despite knowledge of post-acute infection syndromes.Çöcharacterised by similar symptoms that develop after an acute viral, bacterial, or parasitic infection.Çölong COVID was not acknowledged by the medical and scientific community until almost 6 months after the start of the pandemic. This was achieved largely thanks to the persistent advocacy of people with long COVID, who were crucial in drawing attention to their experience and condition. Although it is estimated that at least 145 million people worldwide have long COVID, there is still no clear consensus on how to define or name the condition.

Long COVID is known by different names, such as post-COVID-19 condition, post-COVID-19 syndrome, or post-COVID conditions, all of which include a wide range of persistent or new symptoms that develop after SARS-CoV-2 infection and last for typically more than 12 weeks to months and up to 2 years, so far. The most common symptoms can be broadly categorised into neurological symptoms (fatigue, brain fog, and headache), respiratory symptoms (chest pain and shortness of breath), and diverse symptoms (heart palpitations and muscle pain). These symptoms are often debilitating enough to leave patients unable to work and are similar to those of Myalgic encephalomyelitis/chronic fatigue syndrome, and those triggered after infections with Ebola virus, poliovirus, borrelia (Lyme Disease), and even SARS-CoV, which fall under the well-known concept of post-acute infection syndrome.

Post-acute infection syndromes have always been neglected, hindering the development of diagnostic or treatment tools. However, the recent amount of attention and resources allocated to long COVID could present the perfect opportunity for breakthroughs in the treatment of post-acute infection syndromes. At present, at least 17 million people have long COVID in the WHO European region, in addition to the unknown number of people with other post-acute infection syndromes. Thus, post-acute infection syndromes could pose a substantial public health burden in the near future if appropriate measures are not immediately taken to provide better diagnostic care, treatment, and clinical infrastructure. Therefore, governments and the medical and scientific community must commit to funding and accelerating research to develop targeted and effective treatments for post-acute infection syndromes.

The development of diagnostic markers and effective therapeutic options for people with post-acute infection syndromes pose three major challenges. The first challenge is that the pathophysiology of such syndromes is not clearly understood. Different hypotheses have emerged, including chronic inflammation, autoimmune response, permanent tissue damage, dysregulation of the microbiota.Çôgut.Çôbrain axis, and pathogen reservoirs. Scientists consider that these mechanisms are not mutually exclusive but could coexist or be pronounced with varying intensity depending on the pathogen. The second challenge for researchers of long COVID is that there is no consensus on a definition and on the categorisation of patients due to the heterogeneity of the symptoms, which prevents their correct inclusion in observational or interventional studies. The third challenge is that no evidence-based treatment options exist at present for long COVID, resulting in the use of a symptom management approach, which mainly includes medical assessment and referral to the appropriate specialist, physical and/or psychological therapy. So far, 36 registered randomised trials are underway, but some have small sample sizes or do not include control groups, which could reduce the applicability of their results. Fortunately, some larger trials are being conducted, for example, the STIMULATE-ICP consortium will test several drugs approved for other diseases and that could be repurposed, such as antihistamines, rivaroxaban, and colchicine. In addition to addressing these challenges, it is also fundamental to take a patient-centred approach by incorporating the feedback and experiences of people with long COVID and other post-acute infection syndromes to better understand these conditions.

Considering that COVID-19 cases are predicted to increase in the coming winter months and vaccination against SARS-CoV-2 lowers the risk of long COVID after infection by only 15%, it is absolutely crucial that governments implement a public health strategy to provide care and support for people with long COVID. The WHO Regional Office for Europe has launched a COVID-19 containment strategy, which focuses on community surveillance, case detection, access to oral antivirals, vaccination roll-out, and mental health promotion. However, these efforts might not be sufficient to prevent new infections and hence new cases of long COVID. At present, the most effective way to prevent long COVID is to prevent SARS-CoV-2 infections by vaccination and non-pharmaceutical interventions.Çöie, wearing masks, avoiding crowded or closed places, and self-isolating at the first sign of infection.

The emergence of long COVID has reiterated the low importance attributed to post-acute infection syndromes by doctors and the scientific community and has also highlighted a considerable gap in scientific knowledge. Fortunately, the initial apathetic response to long COVID evolved remarkably quickly, with the allocation of large amounts of funding for research and the establishment of specialised long COVID clinics, which had not been seen before for other post-acute infection syndromes. This spotlight on long COVID stands as an opportunity to shed light on other known post-acute infection syndromes, but also to set an example for future infectious outbreaks.

